# Investigating the Limits of Predictability of Magnetic Resonance Imaging-Based Mathematical Models of Tumor Growth

**DOI:** 10.3390/cancers17203361

**Published:** 2025-10-18

**Authors:** Megan F. LaMonica, Thomas E. Yankeelov, David A. Hormuth

**Affiliations:** 1 Department of Biomedical Engineering, The University of Texas at Austin, Austin, TX 78712, USA; mlamonica@utexas.edu (M.F.L.); thomas.yankeelov@utexas.edu (T.E.Y.); 2 Oden Institute for Computational Engineering and Sciences, The University of Texas at Austin, Austin, TX 78712, USA; 3 Department of Diagnostic Medicine, The University of Texas at Austin, Austin, TX 78712, USA; 4 Livestrong Cancer Institutes, The University of Texas at Austin, Austin, TX 78712, USA; 5 Departments of Imaging Physics, The University of Texas M.D. Anderson Cancer Center, Houston, TX 77030, USA

**Keywords:** computational oncology, prediction, cancer, uncertainty, tumor forecasting

## Abstract

Background/Objectives: We provide a framework for determining how far into the future the spatiotemporal dynamics of tumor growth can be accurately predicted using routinely available magnetic resonance imaging (MRI) data. Our analysis is applied to a coupled set of reaction-diffusion equations describing the spatiotemporal development of tumor cellularity and vascularity, initialized and constrained with diffusion-weighted (DW) and dynamic contrast-enhanced (DCE) MRI data, respectively. Methods: Motivated by experimentally acquired murine glioma data, the rat brain serves as the computational domain within which we seed an in silico tumor. We generate a set of 13 virtual tumors defined by different combinations of model parameters. The first parameter combination was selected as it generated a tumor with a necrotic core during our simulated ten-day experiment. We then tested 12 additional parameter combinations to study a range of high and low tumor cell proliferation and diffusion values. Each tumor is grown for ten days via our model system to establish “ground truth” spatiotemporal tumor dynamics with an infinite signal-to-noise ratio (SNR). We then systematically reduce the quality of the imaging data by decreasing the SNR, downsampling the spatial resolution (SR), and decreasing the sampling frequency, our proxy for reduced temporal resolution (TR). With each decrement in image quality, we assess the accuracy of the calibration and subsequent prediction by comparing it to the corresponding ground truth data using the concordance correlation coefficient (CCC) for both tumor and vasculature volume fractions, as well as the Dice similarity coefficient for tumor volume fraction. Results: All tumor CCC and Dice scores for each of the 13 virtual tumors are >0.9 regardless of the SNR/SR/TR combination. Vasculature CCC scores with any SR/TR combination are >0.9 provided the SNR ≥ 80 for all virtual tumors; for the special case of high-proliferating tumors (i.e., proliferation > 0.0263 day^−1^), any SR/TR combination yields CCC and Dice scores > 0.9 provided the SNR ≥ 40. Conclusions: Our systematic evaluation demonstrates that reaction-diffusion models can maintain acceptable longitudinal prediction accuracy—especially for tumor predictions—despite limitations in the quality and quantity of experimental data.

## Simple Summary

Understanding how brain tumors grow over time is important for improving and personalizing treatment. In this study, we used mathematical models to simulate tumor growth in the brain and tested how well these models can predict future changes using standard MRI scans. We looked at how different levels of image quality—such as noise, detail, and timing—affect the accuracy of these predictions. Our results show that even when the MRI data is not perfect, the models can still make reliable predictions about tumor growth, especially if the images are not noisy. This research may help guide the design of future imaging experiments to make useable predictions, potentially reducing the need for frequent or high-quality scans while still providing valuable insights into tumor behavior.

## 1. Introduction

High-grade gliomas, including glioblastoma (GBM), are glial cell tumors found in the brain and spinal cord. Unfortunately, even with an aggressive treatment strategy that includes surgical resection followed by radiation therapy and chemotherapy, tumors almost invariably recur, resulting in a median survival after diagnosis of less than two years [1]. Thus, new ways to guide and improve interventions are desperately needed. One potential way to improve treatment strategies is to develop medical imaging-informed mathematical models that can accurately predict tumor response to treatment [2,3,4], thereby providing insights into how to optimize interventions. However, it is unclear how limitations in image quality, specifically signal-to-noise ratio (SNR), spatial resolution (SR), and temporal resolution (TR), may influence the accuracy of image-informed models. Therefore, our question is: what quantity and quality of imaging data are sufficient for accurately predicting the spatiotemporal growth and treatment response of a tumor? In the present study, we investigate this question for a magnetic resonance imaging (MRI)-informed, mechanically coupled reaction-diffusion model of glioma growth that we have previously established [5,6,7,8]. We will address this question in the pre-clinical setting, where we are not data-limited and can systematically adjust SNR, SR, and TR in a well-defined and well-controlled manner.

We are not the first to evaluate how image quality affects the calibration of model parameters and model predictions; it is an area of active research across mathematical models and tumor types. For example, Rutter et al. evaluated a reaction-diffusion model of GBM with parameter probability distributions for diffusion and proliferation parameters [2]. They assessed the recoverability of the original parameter distributions given noisy synthetic data and found that up to a noise level of 5%, the parameters were recoverable using the Prokhorov metric framework for inverse problems [2]. Phillips et al. developed a family of MRI-informed breast cancer models to predict patient-specific responses to neoadjuvant therapy and then evaluated the impact of parameter uncertainty on model predictions. They determined that within clinically relevant levels of noise (i.e., 10%), it was still possible to obtain accurate model predictions of patient therapy response with an area under the receiver operating characteristic curve of 0.85 [3]. In a similar fashion, Hiremath et al. evaluated model and parameter identifiability under noisy conditions in an MRI-informed GBM model applied to clinical data collected before, during, and after radiation therapy [4]. Tumor growth was modeled using a two-species model of enhancing and non-enhancing tumors, which can be personalized to longitudinal MRI data. They observed median errors in model parameters ranging from 0.04% to 72.96% when up to 15% noise was added to the measurement.

The impact of measurement and model uncertainty on treatment optimization has also been investigated in the clinical setting. In an effort to develop personalized GBM treatment with an MRI-informed reaction diffusion model of GBM, Le et al. evaluated how uncertainty in model parameters and MRI segmentations affected personalized intensity-modulated radiotherapy (IMRT) plans for two patients [5]. The final IMRT plans that considered uncertainty recommended distributing a higher amount of the total radiotherapy dose near progressive tumor edges and a lower amount near the brainstem than plans that did not take uncertainty into account. The difference in radiation plans suggests that uncertainty analysis warrants further investigation because it could facilitate better-informed treatment plans [5]. Hawkins-Daarud et al. [6] evaluated how uncertainty in the parameters, tumor size, and time between exams affects the uncertainty in “Days Gained”, a metric they define as the number of days a patient gains before tumor growth progresses to where it would have been without treatment. They found that parameters related to the time between two pre-treatment images and the tumor growth kinetics most affected the uncertainty in their Days Gained metric. However, the authors noted that a better understanding of the uncertainty in the Days Gained metric is important for establishing its clinical utility, again highlighting the importance of uncertainty analysis in mathematical oncology.

To contribute to the growing body of work on uncertainty quantification in image-based modeling, we conducted 54 simulation experiments using varying combinations of SNR, SR, and TR. These experiments were applied to 13 distinct in silico tumors, each generated by systematically varying model parameters. The in silico tumors were generated using a coupled model of tumor and vasculature dynamics that captures key biological processes, including tumor growth, angiogenesis, and vessel regression. We then determine the accuracy of the model calibrations by computing the percent error between the known and calibrated parameter values as a function of SNR, SR, and TR. Model prediction accuracy is assessed with the Dice similarity coefficient to assess global tumor volume agreement, and the concordance correlation coefficient (CCC) to assess the local voxel-wise agreement. The goal of this study is to determine what quantity and quality of MRI data is needed for reliable model parameter calibration and prediction, with the aim of establishing practical guidelines for achieving a desired level of accuracy given the inherent limitations in pre-clinical imaging data.

## 2. Materials and Methods

### 2.1. Biophysical Model of Tumor Growth and Angiogenesis

We employ a two-species, reaction-diffusion model of tumor growth and angiogenesis which we have previously established [7]. Equations (1) and (2) describe the spatiotemporal evolution of the tumor, *N_t_*(***x***,*t*), and vasculature, *N_v_*(***x***,*t*), volume fractions, respectively:

(1) $$\begin{array}{c} \frac{\partial N_{t}\left(x,t\right)}{\partial t}=\nabla \cdot \left(D_{t}\left(x,t\right)\cdot \left(\left(1-\frac{N_{v}\left(x,t\right)}{\theta_{tot}\left(x,t\right)}\right)\nabla N_{t}\left(x,t\right)\right)+\frac{N_{t}\left(x,t\right)}{\theta_{tot}\left(x,t\right)}\nabla N_{v}\left(x,t\right)\right)\\ +k_{t}\left(x\right)N_{t}\left(x,t\right)\left(1-\frac{N_{t}\left(x,t\right)}{\theta_{tot}(x,t)}\right) \end{array}$$

(2) $$\begin{array}{c} \frac{\partial N_{v}\left(x,t\right)}{\partial t}=\nabla \cdot \left(D_{v}\left(x,t\right)\left(\left(1-\frac{N_{t}\left(x,t\right)}{\theta_{tot}\left(x,t\right)}\right)\nabla N_{v}\left(x,t\right)\right)+\frac{N_{v}\left(x,t\right)}{\theta_{tot}\left(x,t\right)}\nabla N_{t}\left(x,t\right)\right)+\\ k_{v}\left(x\right)N_{v}\left(x,t\right)\left(1-\frac{N_{v}\left(x,t\right)}{\theta_{tot}\left(x,t\right)}\right)d(x)-k_{d,v}N_{v}\left(x,t\right)\left(1-d(x)\right), \end{array}$$

where

(3) $$\theta_{tot}(\bar{x},t)=\left\{\begin{array}{c} \theta_{min\;}+N_{v}(x,t)\left(\frac{{\theta_{max\;}-\theta }_{min\;}}{N_{v,\;\;thresh}}\right),\;\;N_{v}(x,t)\;<\;N_{v,\;\mathrm{thresh}}\\ \theta_{max\;},\;\;\;\;\;\;\;\;\;\;\;\;\;\;\;\;\;\;\;\;\;\;\;\;N_{v}\;(x,t)\geq \;N_{v,\;\mathrm{thresh}} \end{array}\right..$$

where *D_t_*(***x**,t*) and *D_v_*(***x**,t*) represent the random diffusion of tumor cells and vasculature at position ***x*** and time *t*, respectively, and *k_t_*(***x***) and *k_v_*(***x***,*t*) are the proliferation rates for the tumor cells and vasculature, respectively. Note, a non-linear, cross-diffusion term is used to account for the interaction of multiple species [8]. The growth is restricted by a physical carrying capacity, *θ_tot_*, described in Equation (3); it is the sum of the tumor cell and vasculature carrying capacities. *q_min_* and *q_max_* are the minimum and maximum voxel carrying capacities, respectively, and *N_v_*_,_*_thresh_* is the minimum fraction of vasculature needed to support the maximum tumor cell carrying capacity. Biologically, it represents the maximum number of tumor cells that can be supported in a given area, depending on the vasculature present. Angiogenesis and vessel regression are spatially informed via *d*(***x***), which is the normalized distance to the periphery of the tumor. *d*(***x***) ranges from 1 (i.e., a voxel at the periphery of the tumor) to 0 (i.e., a voxel furthest from the periphery). *k_d_*_,_*_v_* is the vasculature death rate. (See Table 1 for a listing of all model parameters.)

The two diffusion terms in Equations (1) and (2) are coupled to the mechanical properties of the surrounding tissue via Equation (4):

(4) $$D_{t}\left(x,t\right)=D_{t,0}\exp\left(-\lambda \sigma_{vm}\left(x,t\right)\right),$$

where λ is an empirically derived constant that describes the degree to which tumor cell diffusion is impacted by mechanical stress, and *D_t_*_,0_ is the tumor cell diffusion coefficient in the absence of mechanical restrictions. Equation (4) is applied in a similar fashion for *D_v_* (the vascular diffusion coefficient), while *D_t_*_,0_ and *D_v_*_,0_ (vascular diffusion coefficient in the absence of mechanical restrictions) are both assigned as global (i.e., spatially homogenous) parameters. The von Mises stress, *s_vm_*(*x*,*t*), explicitly accounts for spatial differences in the mechanical stress that the tumor experiences. More specifically, the stress depends on the shear modulus of the brain environment, where a higher shear modulus means a more rigid material; in particular, we assume white matter has a greater shear modulus (G = 800 Pa) than gray matter (G = 466 Pa) [9]. We assume linear elastic isotropic mechanical equilibrium (Equation (5)), which states that a gradient of tumor cells exerts a force (with coupling constant *l_f_*) on the environment, and the environment exerts stress, σ, on the tumor in return:

(5) $$\nabla \cdot \sigma =\;\lambda_{f}\nabla N_{t}\left(x,t\right).$$

Equation (5) is solved to determine the local normal and shear stresses, which are then used to compute the von Mises stress, a measure of the effective stress that a tumor experiences in all directions from the surrounding brain tissue. Poisson’s ratio *n* is the ratio of lateral to longitudinal strain. The stress relates to strain per Hooke’s Law for linear elastic isotropic materials:

(6) $$\left[\begin{array}{c} \sigma_{xx}\\ \sigma_{yy}\\ \sigma_{zz}\\ \sigma_{xy}\\ \sigma_{xz}\\ \sigma_{yz} \end{array}\right]=\frac{2G}{1-2v}\left[\begin{array}{cccccc} 1-\nu & v & v & 0 & 0 & 0\\ v & 1-\nu & v & 0 & 0 & 0\\ v & v & 1-\nu & 0 & 0 & 0\\ 0 & 0 & 0 & 1-2\nu & 0 & 0\\ 0 & 0 & 0 & 0 & 1-2\nu & 0\\ 0 & 0 & 0 & 0 & 0 & 1-2v \end{array}\right]\left[\begin{array}{c} \epsilon_{xx}\\ \epsilon_{yy}\\ \epsilon_{zz}\\ \epsilon_{xy}\\ \epsilon_{xz}\\ \epsilon_{yz} \end{array}\right].$$

**Figure 1:**
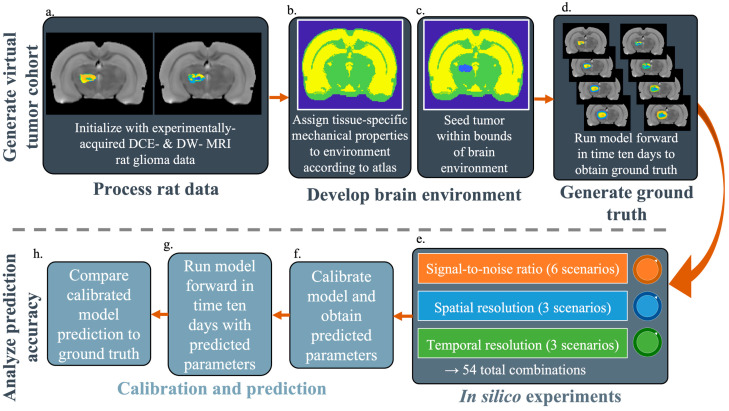
Methods overview. The model is initialized with experimentally acquired DCE- and DW-MRI rat glioma data (**a**) seeded into a brain environment with defined mechanical properties (**b**,**c**). The model is then run forward to generate noiseless ground truth data (**d**). To generate a ground truth tumor, the spatial resolution (SR) and temporal resolution (TR) were set to maximum practical values (0.008 mm^3^ and five calibration timepoints, respectively) with an infinite signal-to-noise ratio (SNR), and the model was then run ten days into the future (**e**). To generate in silico experimental data, noise is added to the day 1 ground truth data, and the data are downsampled to degrade the SNR and SR, respectively. The TR is degraded during model calibration (**f**) by using a nonzero weight for only the desired timepoints (i.e., a subset of the entire time course). The model is then run forward with the calibrated parameters to yield a prediction of tumor growth (**g**). Finally, the predictive accuracy at each SNR, SR, and TR is compared against the ground truth data at day 10 (**h**)

We implement the model (i.e., Equations (1)–(6)) in MATLAB 2022a (MathWorks, Natick, MA, USA) using the 3D finite difference method. Zero flux boundary conditions were employed for *N_t_*(***x***,*t*) and *N_v_*(***x***,*t*). For the biomechanical model we assume zero displacement in the direction normal to the boundary, while displacement was allowed to be non-zero tangential to the boundary. For each experiment, the domain was discretized to match the spatial resolutions of the selected SR. A time step of 0.01 days was selected to maintain numerical stability over the expected range of diffusion coefficients. Full details on the implementation of the mathematical model are presented in [9]. Throughout the Materials and Method section, we encourage the reader to refer to Figure 1 which provides a schematic of the overall approach.

### 2.2. Generate Virtual Tumor Cohort for In Silico Experiments

We first generate 13 in silico tumors at a range of SRs and the best-case SNR and TR to provide a ground truth for comparing model calibrations and predictions made using data with lower SNR and TR. Dice similarity coefficients and CCCs between each SR’s ground truth and the lower-quality test cases are used to quantify reductions in model prediction accuracy. The 13 different sets of model parameters allow us to cover a range of potential tumor growth behaviors, mimicking the variety observed in in vivo settings. To achieve this goal, we uniformly sample parameters by taking the original tumor parameters as the center of parameter space and investigating combinations of diffusion and proliferation for both the tumor and vasculature species based on ±25%, ±50%, and ±75% of those original values. The upper limit of ±75% was selected based on the model parameters from our previous study [7], where we observed the following variations from the mean across the animal cohort: $k_{t}$ ranged from −29.8% to 73.8%, $D_{t}$ ranged from −61.5% to 55.7%, $D_{v}$ ranged from −65.9% to 49.0%, and $k_{v}$ ranged from −67.8% to 71.1%. The original parameter values are 0.0263 mm^2^/day, 0.0100 mm^2^/day, 0.45 day^−1^, and 0.25 day^−1^ for *D_t_*, *D_v_*, *k_t_*, and *k_v_*, respectively (see Figure S1).

#### 2.2.1. Experimental Data Used to Initialize the Simulations

The murine data used to initialize the model are diffusion-weighted (DW-) MRI and dynamic contrast-enhanced (DCE-) MRI data that were previously acquired [10] and approved by the appropriate Institutional Animal Care and Use Committee. The data are from a female Wistar rat inoculated with 10^5^ C6 glioma cells via stereotactic injection. The MR images were obtained using a 9.4 Tesla horizontal bore magnet (Agilent, Santa Clara, CA, USA) and 38 mm Litz quadrature coil (Doty Scientific, Columbia, SC, USA). The images were acquired over a 32 × 32 × 16 mm^3^ field of view that was sampled with a 128 × 128 × 16 matrix. *N_t_*(***x***,*t*) was estimated from the apparent diffusion coefficient, *ADC*(***x***,*t*), values via Equation (7):

(7) $$N_{t}(x,t)=\;\theta_{max}\left(\frac{{ADC}_{w}-ADC(x,t)}{{ADC}_{w}-{ADC}_{min}}\right),$$

where *q_max_* is the maximum tumor cell carrying capacity, *ADC_w_* is the *ADC* of free water at 37 °C (approximately 3 × 10^−3^ mm^2^/s [11]), and *ADC_min_* is the minimum ADC value within the field-of-view. The DCE-MRI data were used to estimate *N_v_*(***x***,*t*) according to the following equation [12]:

(8) $$N_{v}\left(x,t\right)=\;\int_{0}^{60}\frac{C(x,t)}{AIF(t)}dt,$$

where *C*(***x***,*t*) is the time-course of the concentration of contrast agent for the first 60 s after injection for the voxel at position *x*, and the *AIF*(*t*) is the arterial input function [13], which is the concentration of contrast agent in a voxel with a blood volume fraction of 1 (i.e., 100% blood vessel) over the measured time course.

The 3-dimensional matrices of *N_t_*(***x***,*t*) and *N_v_*(***x***,*t*) resulting from the above manipulations were translated so that the tumor region of interest was in the center of the computational (brain) domain to serve as the initial conditions for all 13 in silico tumors. As each in silico tumor exhibits tumor growth properties that differ from those of the animal used to generate the realistic initial conditions, no comparisons will be made longitudinally between the 13 in silico tumors and the in vivo tumor.

#### 2.2.2. Establishing the Computational Domain

The brain tumor environment is generated using *T*_2_-weighted data from the Valdés-Hernández rat brain atlas [14]. K-means clustering of the Valdés-Hernández data was used to segment the brain into two major regions: white matter and gray matter in MATLAB 2022a. As described above in Section 2.1, the locations of the various tissue types are used to inform the mechanically coupled diffusion terms, as outlined in Equations (4)–(6). Once the brain domain is generated, a tumor is seeded near its center using experimentally acquired DCE- and DW-MRI rat glioma data [12]. We chose to place the tumor at the center of the computational brain rather than off-center (as is typically performed in vivo) to maximize the amount of space that the simulated tumor could grow into over 10 days.

#### 2.2.3. Generating SR-Specific Ground Truth Tumors to Compare Against the Model Predictions

We grow a noiseless ground truth tumor for each SR using selected initial conditions and parameter values. To generate ground truth for each SR, the spatial dimensions of the DW- and DCE-MRI data described in Section 2.2.1 are down-sampled (or up-sampled) using MATLAB’s ‘imresize3’. Then Equations (1)–(4) are run forward in time to yield a ten-day time course of the spatiotemporal dynamics of the tumor cell and vasculature (i.e., *N_t_*(***x***,*t*) and *N_v_*(***x***,*t*), respectively). The resulting simulated data for a given SR comprises the ground truth against which subsequent predictions (made using reduced TR and SNR) are compared. This process was repeated for each of the 13 combinations of model parameters, yielding 13 different tumors. Tumors were grown for ten days to match the duration of the original rat experiments [10].

### 2.3. Quantifying the Accuracy of Model Predictions for Specific SNR, SR, and TR Combinations

#### 2.3.1. In Silico Experiments

For each in silico experiment, we select a combination of SNR, SR, and TR (see Table 2). Given the ground truth data constructed as described in the previous section, the SNR is reduced to values of 5, 10, 20, 40, 80, or 160 by multiplying the data by a random normal distribution with a mean of 1 and a standard deviation of 1/SNR using MATLAB’s ‘random’ function. The SR is down-sampled from the highest resolution voxel volume of 0.008 mm^3^ to voxel volumes of 0.063 mm^3^ or 0.50 mm^3^ using MATLAB’s ‘imresize3′ function (as described in Section 2.2.3). The voxel size is increased by the same factor in every dimension. Differences in TR are accounted for in the calibration step described in the next section.

#### 2.3.2. Calibration and Prediction

We calibrate the model described by Equations (1)–(4) using the Levenberg–Marquardt method [9] to a subset of the complete time course data. The TR is varied by adjusting the number of imaging visits used to calibrate the model parameters. We calibrate using either days 1 and 5, days 1, 3, and 5, or days 1 through 5. Thus, we obtain one set of four estimated model parameters (i.e., *D_t_*, *k_t_*, *D_v_*, and *k_v_*) for each combination of SNR, SR, and TR (a total of 54 combinations; see Table 2). The estimated parameters are then used to simulate tumor growth for each tumor out to day 10. We initially test a tumor with model parameters set to 0.0263 mm^2^/days, 0.0100 mm^2^/days, 0.45 days^−1^, and 0.25 days^−1^ for *D_t_*, *D_v_*, *k_t_*, and *k_v_*, respectively. This configuration, tested with *n* = 50 noise replicates, is referred to as the “central tumor”. To assess the sensitivity of model calibration to parameter variation, twelve additional tumors with parameters ±25%, ±50%, and ±75% of the parameter values used for the initial virtual/in silico tumor were also tested. Each of these variant tumors was simulated with *n* = 5 noise replicates. The set of thirteen tumors is referred to as “virtual tumors”. (See Figure S1 for a graphical depiction of the parameters selected for the simulations and a list of the parameters in Table S1.) We test a range of SNR, SR, and TR values that include SNR = 40 and SR = 0.0625 mm^3^, the values achieved for the experimental dataset that inspired the study. All calibrations were performed on an AMD EPYC 7402P (2.8 GHz) CPU with 24 cores, 48 threads, and 128 GB of memory.

### 2.4. Error Quantification and Statistical Analyses

The predicted *N_t_*(***x***,*t*) and *N_v_*(***x***,*t*) (obtained using the calibrated model parameters described in the previous section) are directly compared to the ground truth tumor (the tumor at the same SR, but best-case SNR and TR). At the volume level, we calculate the Dice similarity coefficient to assess the spatial agreement for the predicted and ground truth tumor volume fraction. At the voxel level, we calculate the CCC to assess the agreement between the predicted and ground truth tumor and vasculature volume fractions. The Dice and CCC are calculated at Day 6 (to assess a “short-term” prediction) and Day 10 (“long-term” prediction). Day 6 was chosen because it is one day after the last of the data used to calibrate the model for all TR cases, while Day 10 was selected because it represents the end of the experimental time frame. We tested for statistical differences between error metrics across each in silico experiment using a multi-way analysis of variance (MATLAB’s ‘anovan’). Post hoc comparisons were performed using a multi-way comparison with Bonferroni correction for multiple testing (MATLAB’s ‘multcompare’). The normality of the distributions for all metrics was confirmed using the Lillefors test in MATLAB. The statistically significant differences are reported in the Supplementary Materials. In the main text, we report the results for the “central tumor” (*n* = 50 replicates), while the Supplemental material reports the results for the twelve additional tumors in the parameter space around the central tumor.

## 3. Results

### 3.1. Representative Ground Truth Images

**Figure 2:**
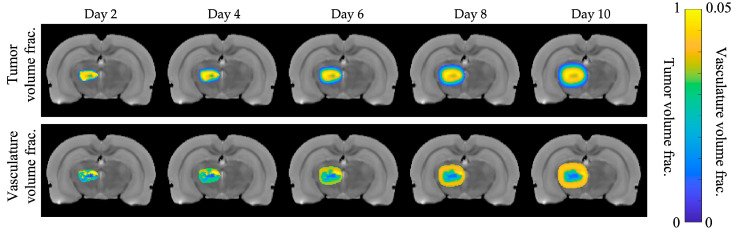
Tumor and vasculature time series. The top and bottom rows show how the tumor cell volume fraction and vascular volume fraction change, respectively, over the course of ten days for best-case SNR = 160 and spatial resolution (SR; voxel volume) of 0.008 mm^3^. The predicted lack of vasculature in the center of the tumor (bottom row) reflects the necrotic core in the tumor volume fraction (top row). Tumor images are overlaid on the anatomical image for reference.

**Figure 3:**
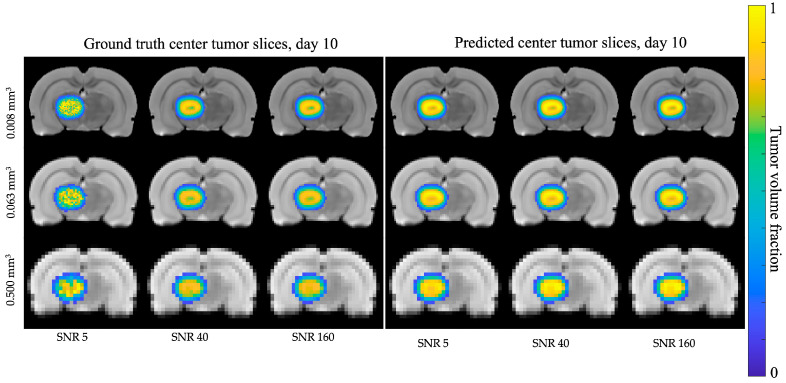
Representative tumor slices. Representative central slices of ground truth tumor data (left panel) and predicted tumor data (right panel) at Day 10 for a low SNR case (SNR = 5), a medium SNR case (SNR = 40), and a high SNR case (SNR = 160) from left to right. From top to bottom, SR is degraded as voxel size increases from 0.008 mm^3^ to 0.063 mm^3^ to 0.500 mm^3^. Note that a balance of SNR and SR is required to adequately capture the necrotic tumor core; in particular, the worst-case SNR and SR cases do not capture this feature. Tumor images are overlaid on the anatomical image for reference.

Figure 2 shows the growth over time for the ground truth tumor grown with the central parameters. Maps of the *N_t_*(***x***,*t*) and *N_v_*(***x***,*t*) for the ground truth tumor at each SR were generated for each of the 13 virtual tumors. Figure 3 presents a representative comparison of ground truth (left panel) versus predicted (right panel) tumor cell maps at day 10 of the simulation (final timepoint) for low-quality (SNR = 5, SR = 0.500 mm^3^), medium-quality (SNR = 40, SR = 0.063 mm^3^), and high-quality (SNR = 160, SR = 0.008 mm^3^) cases. Each of these cases was calibrated using days 1 to 5. Note that not all combinations of SNR and SR were able to accurately reproduce the necrotic core. At an SNR of 5 and an SR of 0.500 mm^3^, the necrotic core is particularly difficult to observe, especially when compared to the best case of an SNR of 160 and an SR of 0.008 mm^3^.

### 3.2. Error in Estimated Parameters

**Figure 4:**
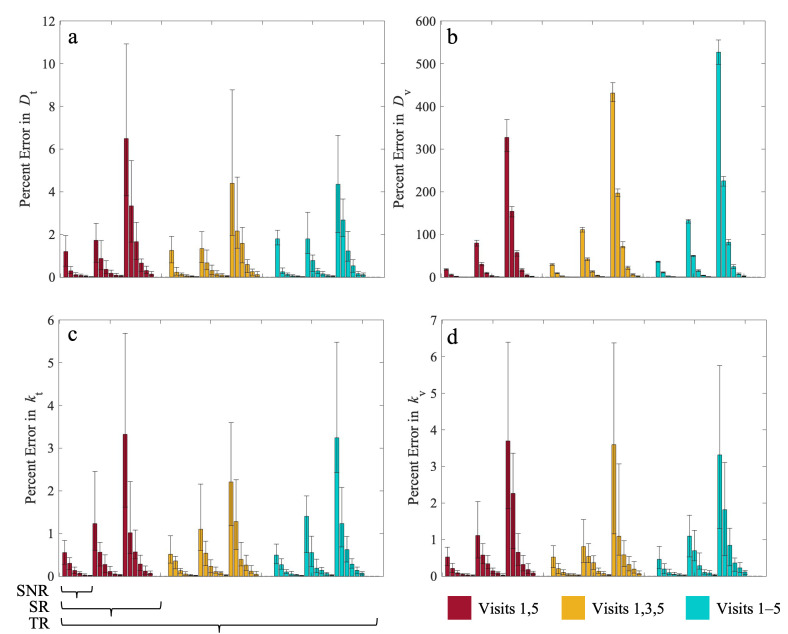
Influence of temporal resolution, spatial resolution, and SNR on parameter error. Median (±25%) percent error (PE) for proliferation rates, panels (**a**,**b**), and diffusion coefficients, panels (**c**,**d**), are shown for all 54 tested in silico experiments. The bar plots are organized as indicated by the bars below panel c, corresponding to the TR, SR, and SNR ranges listed in the Methods section. Each panel is first divided by the three TRs represented by the red, yellow, and blue bar plots. Within each TR group, the remaining bars are sorted by the three SRs. Lastly, within each TR and SR combination, the bars are sorted by the six SNRs as described in the methods. In general, as SNR increases, the error in predicted parameters decreases regardless of SR or TR. Between SR groups, PE decreases as SR improves. Between TR groups, there is less of a noticeable difference than when comparing SNR or SR. The percent error in *D_v_* (**d**) is two orders of magnitude larger than the error in the other parameters, regardless of the SNR/SR/TR combination.

Figure 4 shows the percent errors in estimated model parameters across all combinations of SNR, SR, and TR for the central tumor. (Please see the supplement for figures corresponding to the twelve other parameter combinations (each with *n* = 5 replicates)). The proliferation rates of tumor cells (*k_t_*) and vasculature (*k_v_*), as well as the diffusion coefficient of tumor cells (*D_t_*), have total percent errors < 8%, regardless of the SNR/SR/TR combination. The vasculature diffusion parameter (*D_v_*) has a percent error two orders of magnitude larger than the other parameters, ranging from 0.10% to 531.37%. However, its error follows the same trend of decreasing percent error as SNR improves. Statistically significant differences are reported in Supplemental Figure S5. Additionally, each in silico experiment is listed in Supplemental Table S2. 

### 3.3. Error in Tumor Growth Predictions

**Figure 5:**
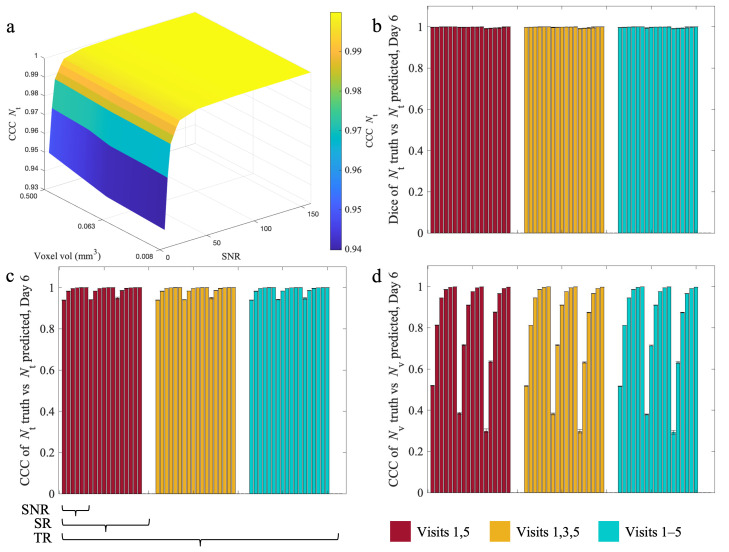
Influence of temporal resolution, spatial resolution, and noise on Dice and CCC at day 6. Median (±25%) Dice and CCC scores at day 6 are shown for all 54 tested in silico experiments. Panel (**a**) is a surface plot showing tumor CCC scores across all SNR and SR for the case where TR is maximized. CCCs improve as SNR improves, but it is >0.9 for all cases. Similar patterns emerge for different TRs. The bar plots (**b**–**d**) are organized by the bars below panel c, corresponding to the TR, SR, and SNR ranges listed in the methods section. Each panel is first divided by the three temporal resolutions represented by the red, yellow, and blue bar plots. Within each TR group, the remaining bars are sorted by the three SRs. Lastly, within each TR and SR combination, the bars are sorted by the six SNR values tested as described in the Methods. In general, Dice (**b**) and CCC are >0.9 or all combinations for both tumor cells (**c**) and vasculature (**d**), except for the CCC of the vasculature at an SNR of 5. There is a slight improvement in Dice and CCC overall as SNR improves across SR and TR.

Figure 5 presents the influence of SNR, SR, and TR on Dice and CCC values by Day 6 of the experiment (that is, one day after the last calibration timepoint on Day 5). Values are shown as median ±25% interquartile ranges. Panels A-C report the prediction results for the tumor volume fraction. Panel A visualizes the tumor CCC scores across all SNR and SR combinations with the maximal TR. While CCC improves across SNR, it is acceptable (i.e., above > 0.9 [15]) for all cases. Panel B reports the Dice similarity, demonstrating Dice values exceeding 0.90 for all combinations of SNR/SR/TR. Panel C is a bar plot showing the information for all TR cases, indicating similar accuracy regardless of TR. For the vasculature, we observed CCCs ranging from 0.30 to 0.99, generally increasing with increased SNR, SR, and TR. Dice and CCC scores for both tumor and vasculature fractions are high (>0.9) for all SNR/SR/TR combinations, with the exception of vasculature CCC at SNR values of 5 and 10. Statistically significant differences are reported in Supplemental Figure S6. 

**Figure 6:**
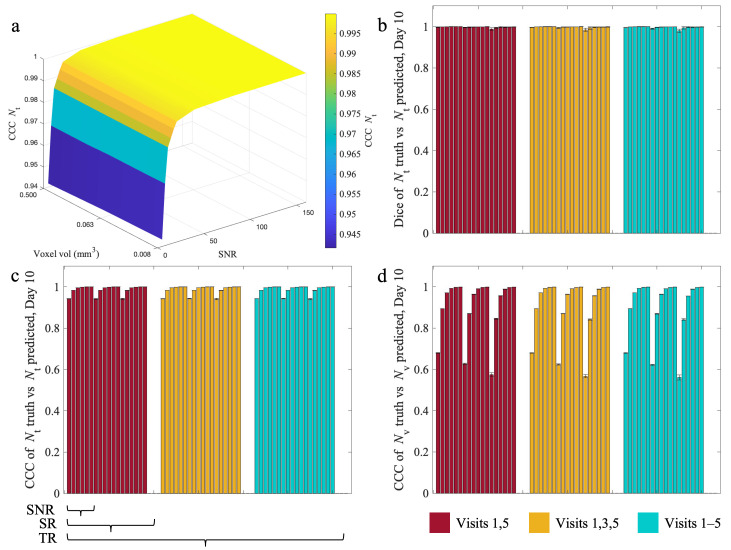
Influence of temporal resolution, spatial resolution, and noise on Dice and CCC at day 10. Median (±25%) Dice and CCC scores at day 10 are shown for all 54 tested in silico experiments. Panel (**a**) is a surface plot showing tumor CCC scores across all SNR and SR for the case where TR is maximized. The bar plots are organized as indicated by the bars below panel c, corresponding to the TR, SR, and SNR ranges listed in the methods section. Each panel is first divided by the three temporal resolutions represented by the red, yellow, and blue bar plots. Within each TR group, the remaining bars are sorted by the three SRs. Lastly, within each TR and SR combination, the bars are sorted by the six SNRs that were tested as described in the methods. In general, Dice (**b**) and CCC are high for all combinations for both tumor cells (**c**) and vasculature (**d**) except for the CCC of the vasculature at an SNR of 5, but this value is higher than that found at the short-term day 6 prediction in Figure 4. There is a slight improvement in Dice and CCC overall as SNR improves across SR and TR.

Figure 6 shows the influence of SNR, SR, and TR on Dice and CCC values by Day 10 of the experiment (that is, the last day of the experiment). Panels A-C report the prediction results for the tumor volume fraction. Panel A provides a surface plot visualization of tumor CCC scores across all SNR and SR combinations, with maximal TR. While CCC improves across SNR, it is high for all cases (>0.9). At the global level, panel B shows a high level of agreement with Dice values greater than 0.9 for all cases. Panel C is a bar plot showing the tumor CCC for all TR, as well, where we see similar accuracy regardless of TR. For the vasculature, panel D reports CCCs ranging from 0.54 to 0.99, generally increasing with SNR, SR, and TR. Dice and CCC scores for both tumor and vasculature fractions are high (>0.9) for all SNR/SR/TR combinations except for vasculature CCC at an SNR of 5 or 10. The Day 10 predictions in Figure 6 are similar to those of Day 6 in Figure 5 (SNR 5: median CCCs of 0.38 for Day 6, 0.62 for Day 10; SNR 10: median CCCs of 0.72 for Day 6, 0.87 for Day 10). Statistically significant differences are reported in Supplemental Figure S6.

**Figure 7:**
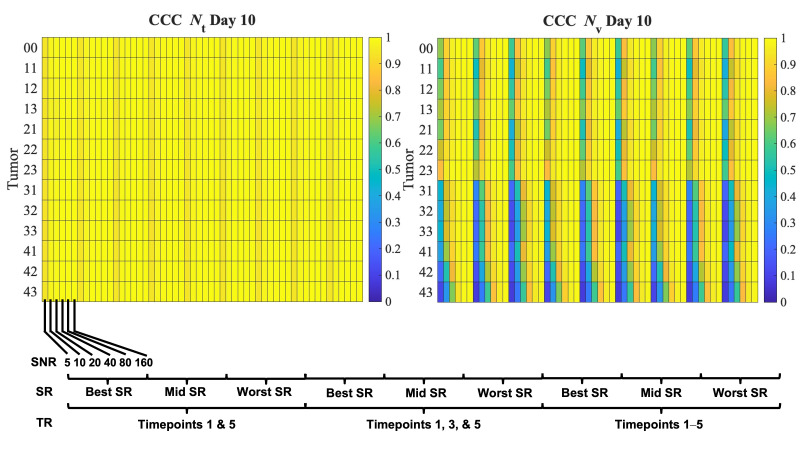
Comparison of SNR, SR, and TR influence on tumor and vasculature CCC across all virtual tumors by day 10. CCC scores at Day 10 are shown for all tumors (along the vertical axis, ID number described in Figure S1 and Table S1) and 54 tested in silico experiments (along the horizontal axis). The heatmaps show how each experiment’s CCC varied for tumor cells and vasculature. The tumor cell CCCs for all virtual tumors and experimental conditions achieved a CCC > 0.9, which demonstrates a high level of accuracy. Tumor CCC scores improved as SNR improved. The vasculature CCC scores were more sensitive to changes in tumor type. Tumors with low proliferation (vertical axis, rows 31–43) had lower vasculature CCC scores than higher-proliferating tumors (vertical axis, rows 11–23) at low SNR levels (SNR 5–20). All virtual tumors achieved sufficient (>0.9) vasculature CCC scores once the SNR reached 80, and all but tumors 42 and 43 in the lowest spatial resolution case achieved a CCC score > 0.9 once the SNR reached 40.

Figure 7 compares the influence of SNR, SR, and TR on tumor and vasculature CCC across all thirteen virtual tumors at day 10. The tumor cell CCCs for all virtual tumors and experimental conditions achieved a CCC > 0.9. As observed for the central tumor, tumor CCC scores improved as SNR improved. The vasculature CCC scores varied most with changes in the virtual tumor. For example, tumors with low proliferation (vertical axis, rows 31–43) had lower vasculature CCC scores (starting at 0.4 for SNR 5–20) than higher-proliferating tumors (vertical axis, rows 11–23), whose CCC scores started at 0.5 for those same SNR cases. All virtual tumors achieved sufficient (>0.9) vasculature CCC scores by an SNR of 80, and all but tumors 42 and 43 in the lowest spatial resolution case achieved a CCC > 0.9 with an SNR of 40. Please see the supplement for a visualization of the tumor parameter space (Figure S1 and Table S1), a comparison of parameter percent error across all virtual tumors (Figure S2), and a comparison of Dice and CCC scores at Days 6 and 10 for all virtual tumors (Figures S3 and S4, respectively). 

## 4. Discussion

We have evaluated a uniformly sampled range of proliferation and diffusion parameters to determine the effect of tumor growth behavior on model prediction uncertainty in response to changes in SNR, SR, and TR. Accurate parameter estimates were achieved for all 54 combinations of SNR, SR, and TR, with no combination exceeding an 8% error for any parameter, except for the *D_v_* parameter. For the SNR combinations, we observed that the error fell sharply as the SNR improved above 40. This finding is consistent with work performed by Xiao et al., whose analysis of parameter estimation in a cancer invasion model found parameter error to be less than 9% [16]. We found that tumors with a tumor proliferation rate greater than 0.45 day^−1^ and vasculature proliferation rate greater than 0.25 day^−1^ had a higher percent error in the *D_t_* and *k_t_* parameters at the lowest spatial resolution compared to other virtual tumors (Figure S2).

We evaluated our model’s predictive accuracy via CCC and Dice scores. We find that all virtual tumors and combinations of SNR/SR/TR gave sufficient tumor (*N_t_*) CCC and Dice values (>0.9) on Days 6 and 10, indicating strong global and local predictions. This was not the case for vasculature CCC scores. Tumors in quadrants 3 and 4 of parameter space (i.e., low proliferation, 25–75% less than the central tumor) had worse vasculature agreement at the voxel level: 49% of their SNR/SR/TR combinations had CCC < 0.9) than tumors in quadrants 1 and 2 (35% of their SNR/SR/TR combinations had CCC < 0.9). An SNR of only 20 was required for all tumors in quadrants 1 and 2 to obtain vasculature CCCs > 0.9, but an SNR of 80 was required for all tumors in quadrants 3 and 4 to achieve the same (CCC > 0.9) (Figure S3). A potential source of the discrepancy between the performance of the vasculature and the tumor model is the differences in magnitude and range of the tumor and vasculature values, which are exacerbated at low SNR. This disparity influences the model calibration, where the combined objective function is dominated by the larger error contribution from the tumor. This can cause the optimization to preferentially select the parameter sets that improve the tumor fit at the expense of the vasculature fit. A potential solution is to implement a weighted objective function or reformulate the problem as a multi-objective optimization to find the set of Pareto optimal solutions [17]. Furthermore, this difference in magnitude also affects the CCC calculation, which requires a detection threshold to be applied first. At low SNR, this thresholding may exclude low-signal voxels and thereby impact the final CCC value. This is not an issue for the tumor as its high signal magnitude makes it more resilient to thresholding effects. A related challenge with the vasculature model is the substantial error observed in *D_v_*. This is most likely due to the model’s low sensitivity to this parameter. A previous Sobol analysis [7,18] of this model determined that *D_v_* had a negligible influence on the model outputs with a Sobol index near 0, while *k_t_*, *k_v_*, and *D_t_* had Sobol indices greater than 0.30. This suggests that the observed error in the vascular diffusion parameter is unlikely to significantly impact the utility of the model in the pre-clinical or clinical setting, as these parameters do not strongly affect the overall predictive accuracy.

The ranges of SNR, SR, and TR investigated in this study are relevant to pre-clinical experiments. In particular, the SNR is strongly dependent on the field strength used for the experiment [19] which is expected to increase with increased field strength. Previous work has demonstrated that anatomical rat brain imaging (e.g., *T*_2_) can achieve an SNR of approximately 30–45 with a 3T scanner and 70–110 with a 7T scanner, depending on particular scan settings and hardware configurations [20]. Similarly, for DW-MRI at 7T, SNR values have been reported to range between 95.0 and 101.8 for voxel volumes between 0.200 mm^3^ and 0.303 mm^3^ [21]. Existing work evaluating spatial resolution in rat brain MRI demonstrates that spatial resolution could be 0.59 mm × 0.59 mm× 1.0 mm or less, which is within the limits of our tested voxel volumes [21]. Our highest temporal resolution of imaging, once every 24 h, is also achievable in the pre-clinical setting; however, we note that repeated anesthetization of animals could disrupt their health and overall well-being, potentially limiting the duration of the study.

A key methodological choice in this study was to evaluate the effects of SR, SNR, and TR on model-derived measures of tumor and vasculature rather than on synthetic (or real) MRI data. This approach was selected to directly investigate the impact of input data quality on parameter calibration under the idealized scenario where the mathematical model accurately represents the underlying biology. From the perspective of uncertainty quantification, this strategy effectively decouples parameter uncertainty (which arises from noisy or limited data) from structural uncertainty, which arises from the model’s own potential inadequacies in representing the real-world system [22,23]. By isolating the analysis to parameter uncertainty, we can establish a foundational understanding of how measurement error propagates through the calibration framework. An alternative approach would involve creating a full in silico imaging pipeline. This would entail generating synthetic DW-MRI and DCE-MRI data from a given set of tumor and vasculature distributions and then exploring how different MRI acquisition settings and hardware choices impact the final parameter estimates after image processing. However, this approach is non-trivial, as realistically synthesizing advanced sequences like DW-MRI and DCE-MRI requires complex, multi-scale biophysical models of water diffusion, vascular permeability, and contrast agent kinetics, along with MRI signal physics simulators. While Jackson et al. [24] have made significant strides towards creating synthetic MRI data from mathematical model outputs using the Bloch equations to simulate *T_2_*-weighted MRI, this methodology is not readily adaptable to advanced sequences such as DW-MRI and DCE-MRI. In the future, this may be more attainable, especially with further development of virtual imaging trial methodology [25], which aims to accelerate the design and optimization of imaging systems and protocols for clinical use.

Our approach utilizes an in silico-generated ground truth tumor with known parameters, which is appropriate when determining error estimates, but does come with a set of limitations. However, in many experimental contexts, the “true parameters” would not be known. Additionally, the tumors in this in silico study grow more regularly than in vivo tumors because they were grown with a selected set of global parameters based on previous studies. Furthermore, there is no consideration of biological factors like genetic mutations that can impact tumor development. While the C6 cell line we use is widely recognized as a suitable choice for animal studies about high-grade gliomas, mutations in isocitrate dehydrogenase status, recognized as an important marker of human GBM prognosis [26,27,28], are not detected in the C6 cell line. Additionally, the C6 cell line does not account for O^6^-methylguanine-DNA methyltransferase (MGMT) methylation status, where a methylated MGMT is associated with resistance to temozolomide in the human clinical setting.

While the primary focus of this manuscript is to provide practical guidelines for pre-clinical experiments, there are some points to consider for translating this approach to the clinical setting. Notably, there are significant differences in the spatial and temporal scales, tumor invasiveness, and heterogeneity in human glioma growth and response. There are two primary limitations of our approach that hinder the direct translation of the model to the clinical setting. First, the C6 glioma model [29] exhibits relatively homogenous growth and response patterns across animals compared to human disease and is less invasive than human gliomas. This limitation could be addressed by performing experiments on a variety of patient-derived xenograft models that better capture the diversity of growth and response parameters and/or tumor growth behaviors (e.g., mass-forming vs. invasive tumors). Second, the mathematical model used in this study is appropriate for mass-forming tumors, such as the C6 glioma, but is not designed to account for the invasive component often observed in human gliomas. To address these challenges in the clinical setting, we employ a two-species model that enhances (most similar to the tumor growth model in this work) and non-enhancing regions of high-grade gliomas, corresponding to mass-forming and invasive disease, respectively. This approach allows us to capture inter-patient heterogeneity in growth, invasion, and response parameters through patient-specific model calibration. When applied to longitudinal clinical data before, during, and after radiotherapy, we have demonstrated that the models and parameters are identifiable [4] and have strong predictive power [30].

The proposed framework has implications for the design of digital twin frameworks [31,32], particularly those utilizing MRI data, such as in brain [30,33,34], breast [35], prostate [26,27], and kidney cancer [36]. This methodology could be adapted to these domains, incorporating site-specific variations in models, treatment protocols, and underlying biological assumptions, to identify optimal imaging strategies that enhance the quality and utility of the information obtained and reduce uncertainty in digital twin predictions [37]. Moreover, as imaging strategies may need to adapt depending on whether a patient is undergoing treatment or being monitored, this framework could be further enhanced by integrating Bayesian information approaches [38,39]. Such an approach would enable a dynamic, data-driven strategy to determine the optimal timing for subsequent imaging based on specific image quality parameters (e.g., SR and SNR) and the uncertainty in the model predictions and parameter estimates. This dynamic methodology has the potential to improve both the efficiency and effectiveness of imaging protocols, ultimately supporting more personalized and adaptive care. Beyond biochemical considerations, it is important to note that a multitude of factors influence image SNR, SR, and TR. Hardware specifications, acquisition protocols, and tissue characteristics impact SNR [19], while SR and TR are affected by voxel size, acquisition speed, and reconstruction techniques. Additionally, the selection of one of these acquisition parameters places limits on what can be selected for the others. Practical constraints such as scanning time, patient comfort, and compliance further limit the selection of imaging parameters. Balancing these interrelated factors is essential, particularly in clinical settings where patient throughput, motion artifacts, and treatment schedules must be considered. Indeed, these factors are regularly considered in the design of new protocols to meet clinical and research needs [40,41]. Additionally, the clinical relevance of longitudinal accuracy varies based on the study’s context; for example, trying to predict the effects of radiation therapy on a 24 h horizon for daily adaptation [42] will not require the same level of longitudinal accuracy as that required for a months-long study. Our study provides one example of how mathematical modeling accuracy varies with SNR, SR, and TR. Extending our methodology will require careful consideration of the practical experimental limitations.

## 5. Conclusions

Our systematic evaluation of the effects of data quality and quantity on model parameter estimation and longitudinal tumor prediction suggests that reaction-diffusion-based models can perform with acceptable longitudinal accuracy even when initialized with data that may have limited SNR, SR, and TR. For the tumor species, *N_t_*, the CCC and Dice scores are acceptable (>0.9) for all tested combinations of SNR, SR, and TR. However, the vasculature species *N_v_* requires an SNR of 40–80 (depending on tumor type) to achieve a sufficient CCC score. Thus, if the SNR is kept above 40, then the predictive accuracy, as quantified by CCC and Dice, should remain sufficient at least 10 days into the future.

## Figures and Tables

**Table 1:** Model parameters and variables.

Parameter or Variable	Description	How Assigned
*N_t_*(***x***,*t*)	fraction of tumor cells per voxel	Initialized at Day 1 with murine DW-MRI data
*N_v_*(***x***,*t*)	fraction of vasculature per voxel	Initialized at Day 1 with murine DCE-MRI data
*D_t_*_,0_	diffusion coefficient of tumor cells	Calibrated
*D_v_*_,0_	diffusion coefficient of vasculature	Calibrated
*k_t_*	proliferation rate of tumor cells	Calibrated
*k_v_*	proliferation rate of vasculature	Calibrated
*θ_min_*	minimum carrying capacity	Set to 0.1
*θ_max_*	maximum carrying capacity	Set to 0.9716
*θ_tot_*	total carrying capacity	Set to *θ_max_* + *θ_min_*
*N_thresh_*	*N_v_* fraction at which max *N_t_* can be supported	Set to 0.022
*k_d_*_,_ *_v_*	Vasculature death rate	Set to 0.125 day^−1^
γ	coefficient representing the impact of von Mises stress on diffusion	Set to 0.25
*d*(***x***,*t*)	Normalized distance to periphery	Calculated
*σ_vm_*	von Mises stress	Calculated

**Table 2:** Image acquisition parameters for in silico experiments.

Acquisition Parameter	Values
Signal-to-noise ratio (SNR)	5, 10, 20, 40, 80, 160
Spatial resolution (SR)	0.008 mm^3^, 0.063 mm^3^, 0.50 mm^3^
Temporal resolution (TR)	5, 3, or 2 timepoints used during calibration

## Data Availability

Data is available from the authors upon request.

## Supplementary Materials

The following supporting information can be downloaded at: https://www.mdpi.com/article/10.3390/cancers17203361/s1. Figure S1: Tumor parameter space, Figure S2: Influence of temporal resolution, spatial resolution, and SNR on parameter error for different virtual tumors, Figure S3: Influence of temporal resolution, spatial resolution, and SNR on CCC and Dice at Day 6 for different virtual tumors. Figure S4: Influence of temporal resolution, spatial resolution, and SNR on CCC and Dice at Day 10 for different virtual tumors. Figure S5: Significant differences observed for model parameters error. Figure S6: Significant differences observed for tumor and vasculature error metrics. Table S1: List of model parameters used for 13 virtual tumors. Table S2: In silico experiment TR, SNR, and SR combinations.

## Abbreviations

The following abbreviations are used in this manuscript:

SNR	Signal-to-noise ratio
SR	Spatial resolution
TR	Temporal resolution
MRI	Magnetic resonance imaging
CCC	Concordance correlation coefficient
