# Supplementary material for: Investigating the Limits of Predictability of Magnetic Resonance Imaging-Based Mathematical Models of Tumor Growth
Source: Cancers (Basel). 2025 Oct 18;17(20):3361. doi: 10.3390/cancers17203361 (PMC12564088; doi:10.3390/cancers17203361)
Supplement: Supplementary file 1 [file cancers-17-03361-s001.zip › cancers-3849986-supplementary.pdf]

Supplemental Material

Investigating the limits of predictability of magnetic resonance imaging-based mathematical models of tumor growth

Megan F. LaMonica[1], Thomas E. Yankeelov[1-5] and David A. Hormuth, II [2,4*]

1 Department of Biomedical Engineering, The University of Texas at Austin; mlamonica@utexas.edu (M.F.L), thomas.yankeelov@utexas.edu (T.E.Y.)
2 Oden Institute for Computational Engineering and Sciences, The University of Texas at Austin; david.hormuth@austin.utexas.edu (D.A.H.)
3 Department of Diagnostic Medicine, The University of Texas at Austin; david.hormuth@austin.utexas.edu (D.A.H.)
4 Livestrong Cancer Institutes, The University of Texas at Austin; david.hormuth@austin.utexas.edu (D.A.H.)
5 Departments of Imaging Physics, The University of Texas M.D. Anderson Cancer Center
* Correspondence: david.hormuth@austin.utexas.edu;

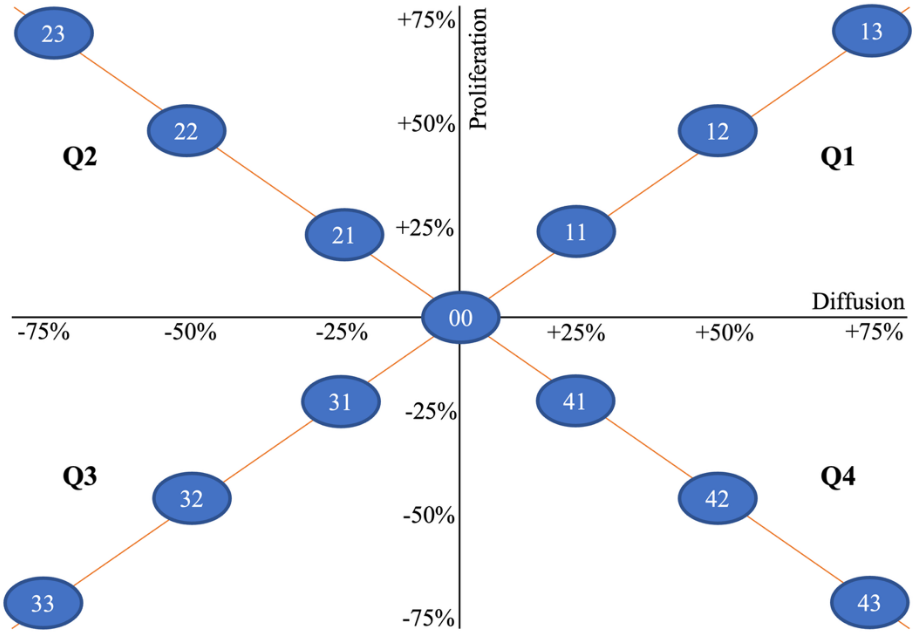

Supplemental Figure 1: Tumor parameter space. *In silico* tumors are named based on the distance from the initial tumor (00) (referred to as the central tumor in the main text). The central true parameters are 0.0263 mm²/days, 0.0100 mm²/days, 0.45 days^{-1}, and 0.25 days^{-1} for D_t, D_v, k_t, and k_v, respectively. The tumors in Quadrants 1 and 2 represent those with higher tumor and vasculature proliferation than the initial tumor. Quadrants 3 and 4 represent tumors with lower tumor and vasculature proliferation than the initial tumor. Similarly, Quadrants 2 and 3 represent tumors with lower tumor and vasculature diffusion than the initial tumor. Quadrants 1 and 4 represent tumors with higher tumor and vasculature diffusion. Distance from the origin represents +/- 25%, +/- 50%, and +/- 75% of initial true parameter values.

Supplemental Table 1: List of model parameters used for 13 virtual tumors

	Parameters			
In silico tumor ID	k_t (day^{-1})	k_v (day^{-1})	D_t (mm^2/day)	D_v (mm^2/day)
**00**	0.4500	0.25	0.0263	0.0100
**11**	0.5625	0.3125	0.0329	0.0125
**12**	0.6750	0.3750	0.0395	0.0150
**13**	0.7875	0.4375	0.0460	0.0175
**21**	0.5625	0.3125	0.0197	0.0075
**22**	0.6750	0.3750	0.0132	0.0050
**23**	0.7875	0.4375	0.0066	0.0025
**31**	0.3375	0.1875	0.0197	0.0075

**32**	0.2250	0.1250	0.0132	0.0050
**33**	0.1125	0.0625	0.0066	0.0025
**41**	0.3375	0.1875	0.0329	0.0125
**42**	0.2250	0.1250	0.0395	0.0150
**43**	0.1125	0.0625	0.0460	0.0175

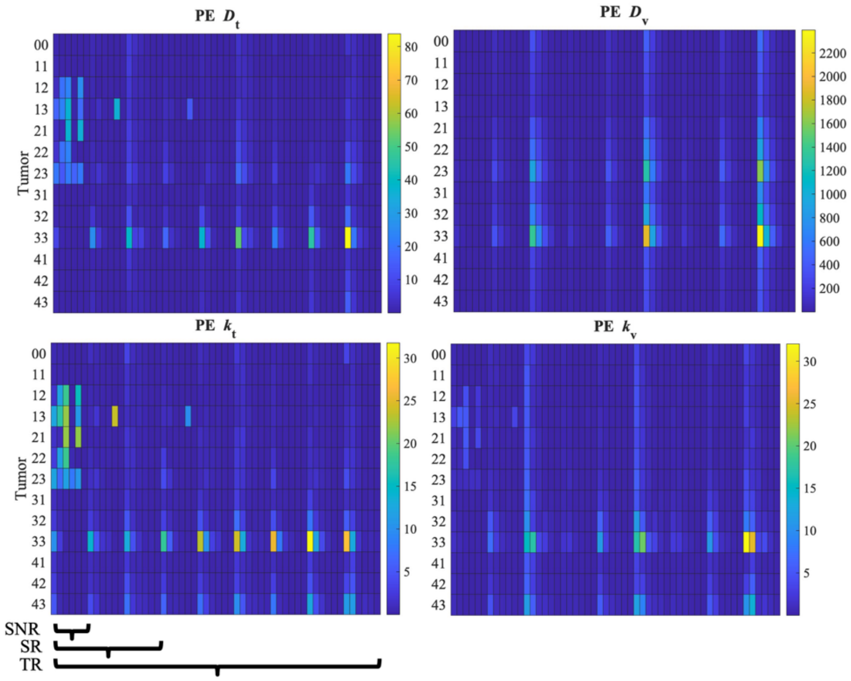

Supplemental Figure 2: Influence of temporal resolution, spatial resolution, and SNR on parameter error for different virtual tumors. Parameter percent error is low for all cases except low SNR cases, and the worst-TR cases for virtual tumors 12, 13,21, 22, and 23. These virtual tumors represent cases with greater proliferation than the original tumor discussed in Figure 3 of the primary manuscript. The percent error in D_v is two orders of magnitude larger than error in the other parameters regardless of combination chosen. Tumor 33 (lowest tested diffusion, lowest tested proliferation) appears to be particularly sensitive to changes in SNR. This could be due to difficulty calibrating parameters associated with little tumor growth.

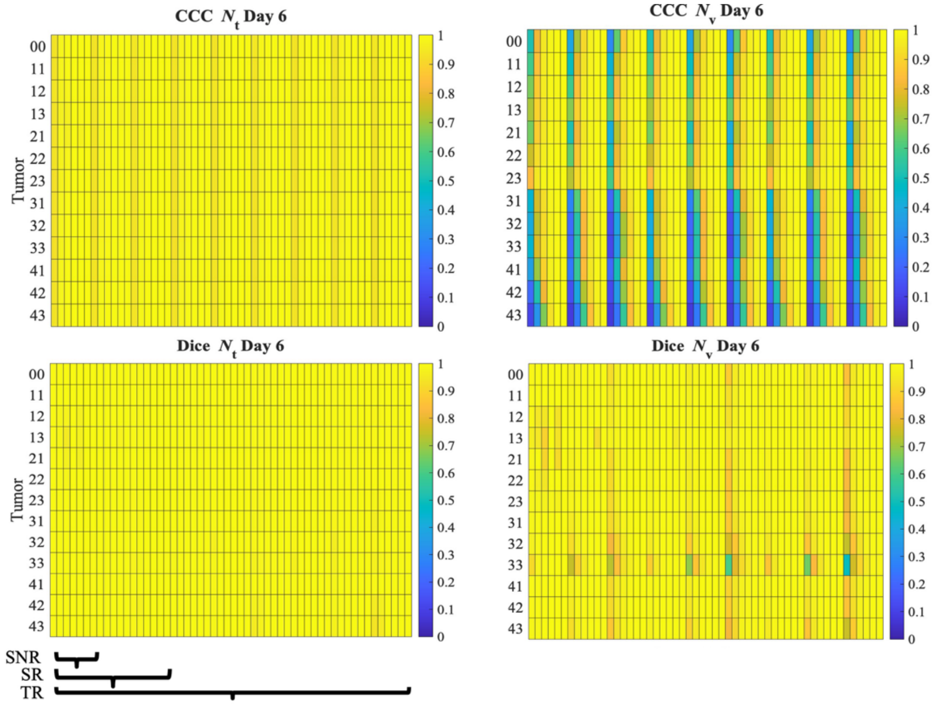

Supplemental Figure 3: Influence of temporal resolution, spatial resolution, and SNR on CCC and Dice at Day 6 for different virtual tumors. Dice and CCC scores quantifying the agreement between predicted and true N_t values are > 0.9 for all combinations and all virtual tumors. Dice and CCC scores quantifying the agreement between the predicted and true N_v values are > 0.9 for all combinations and all virtual tumors once the SNR reaches 20. The CCC metric is more sensitive to changes in SNR than the Dice metric. Virtual tumors with low proliferation (31, 32, 33, 41, 42, and 43) relative to the original tumor in the main manuscript had lower CCC scores than the other virtual tumors when SNR < 20; the low-proliferating tumors required an SNR of at least 20 to achieve sufficient CCC scores, while the higher-proliferating tumors required only an SNR of 10.

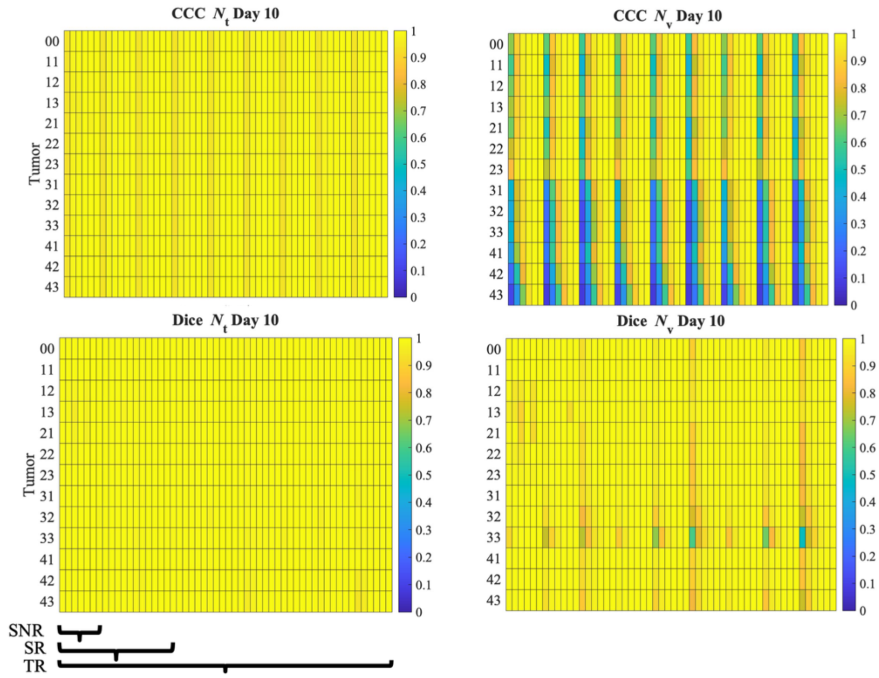

Supplemental Figure 4: Influence of temporal resolution, spatial resolution, and SNR on CCC and Dice at Day 10 for different virtual tumors. Dice and CCC scores pertaining to the tumor species N_t are sufficient (> 0.9) for all combinations and all virtual tumors. Dice and CCC scores pertaining to the vasculature species N_v are sufficient (> 0.9) for all combinations and all virtual tumors above an SNR of 20. The CCC metric is more sensitive to changes in SNR than the Dice metric. Virtual tumors with low proliferation (31, 32, 33, 41, 42, and 43) relative to the original tumor in the main manuscript had lower CCC scores than the other virtual tumors at low SNRs (< 20); the low-proliferating tumors required an SNR of at least 20 to achieve sufficient CCC scores, while the higher-proliferating tumors required only an SNR of 10.

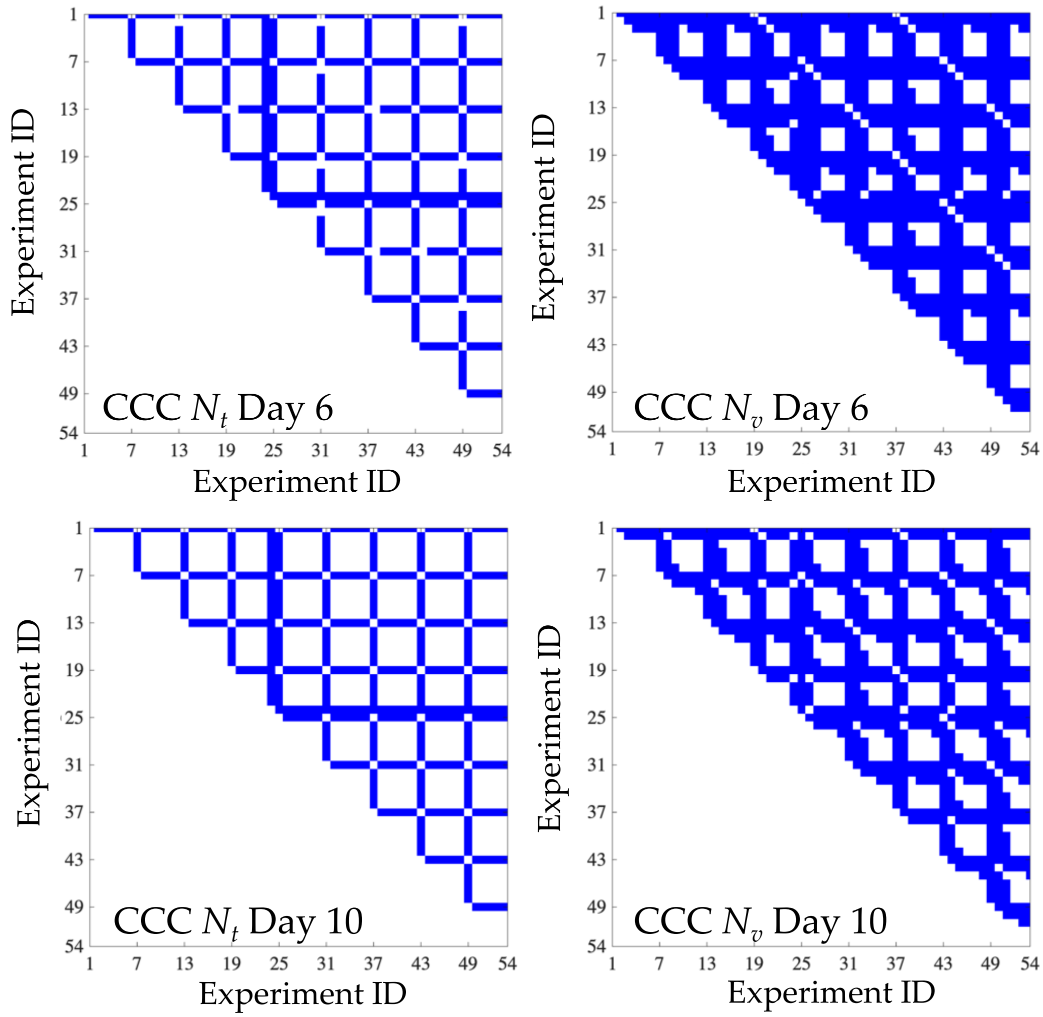

Figure S5: Significant differences observed for model parameters error. The above plots show the comparisons between *in silico* experiments (i.e., variations in SR, SNR, and TR) that yielded statistically significant different results (as indicated in blue) in CCC. Dice for day 6 and 10 was not included as no statistically significant differences were observed. Supplemental Table S2 lists all of the experimental conditions. The statistical analysis, represented by white boxes on each plot, reveals a consistent pattern within different signal-to-noise ratio (SNR) levels. For most experiments, no significant differences were found when comparing the low SNR conditions to each other (small white boxes), nor when comparing the high SNR conditions to each other (large white boxes).

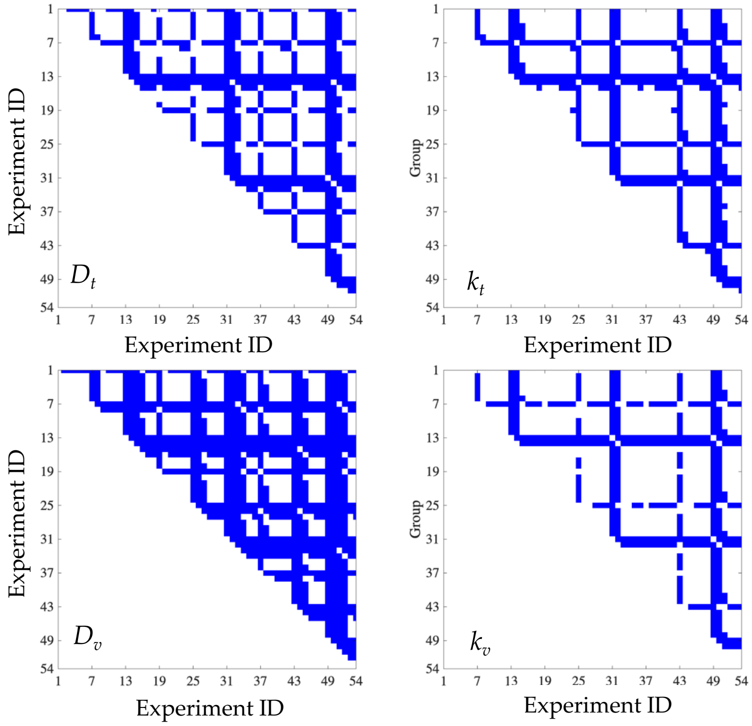

Figure S6: Significant differences observed for tumor and vasculature error metrics. The above plots show the comparisons between *in silico* experiments (i.e., variations in SR, SNR, and TR) that yielded statistically significant different results (as indicated in blue) in parameter estimation error. Supplemental Table S2 lists all of the experimental conditions. The statistical analysis, represented by white boxes on each plot, reveals a consistent pattern within different signal-to-noise ratio (SNR) levels. For most experiments, no significant differences were found when comparing the low SNR conditions to each other (small white boxes), nor when comparing the mid-high SNR conditions to each other (large white boxes).

Supplemental Table S2: In silico experiment TR, SNR, and SR combinations

Experiment ID as a function of TR				
TR (2 time points)	TR (3 time points)	TR (5 time points)	SNR	SR
1	19	37	5	0.50 mm^3
2	20	38	10	0.50 mm^3
3	21	39	20	0.50 mm^3
4	22	40	40	0.50 mm^3
5	23	41	80	0.50 mm^3
6	24	42	160	0.50 mm^3
7	25	43	5	0.063 mm^3
8	26	44	10	0.063 mm^3
9	27	45	20	0.063 mm^3
10	28	46	40	0.063 mm^3
11	29	47	80	0.063 mm^3
12	30	48	160	0.063 mm^3

13	31	49	5	0.008 mm^3
14	32	50	10	0.008 mm^3
15	33	51	20	0.008 mm^3
16	34	52	40	0.008 mm^3
17	35	53	80	0.008 mm^3
18	36	54	160	0.008 mm^3
